# Supplementary material for: The Evolution and Characterization of the RNA Interference Pathways in Lophotrochozoa
Source: Genome Biol Evol. 2024 May 7;16(5):evae098. doi: 10.1093/gbe/evae098 (PMC11114477; doi:10.1093/gbe/evae098)
Supplement: evae098_Supplementary_Data [file evae098_supplementary_data.zip › Supplementary_materials.zip!/selected_miRNAs/NoGe_selected_miRNAs/known_miRNA_families_pdf_files/NMRB01002664.1_Notospermus_geniculatus_strain_Ushimado_scaffold2664,_whole_genome_shotgun_sequence_39911.pdf]

Provisional ID : NMRB01002664.1_Notospermus_geniculatus_strain_Ushimado_scaffold2664,_whole_genome_shotgun_sequence_39911

| | | |
|---|---|---|
| Score total | : | 2359.8 |
| Score for star read(s) | : | 3.9 |
| Score for read counts | : | 2350.9 |
| Score for mfe | : | 2 |
| Score for randfold | : | 0 |
| Score for cons. seed | : | 3 |
| Total read count | : | 4623 |
| Mature read count | : | 4485 |
| Loop read count | : | 0 |
| Star read count | : | 138 |

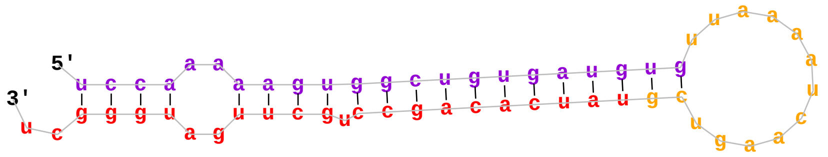

Genome coordinates: NMRB01002664.1 84244-84303

pre-miRNA sequence:
UCCAAAAAGUGGCUGUGAUGUGUCCAAAAAGUGGCUGUGAUGUGUUAAAAUCAAGUCGUAUCACAGCCUGCUUGAUGGGCU

Mature sequence: UAUCACAGCCUGCUUGAUGGGCU

Aligned mature sequence of the *N. geniculatus* pre-miRNA with the top 5 hits from the BLAST search against the pre-miRNA mirGeneDB set:

```
                            ***************
Nge_Mir-2-o18               uaucacagccugcuugaugggcu
Efe-Mir-2-o46_3p            uaucacagccugcuuggauuaug
Lan-Mir-2-o73_3p            uaucacagccugcuuugaugaga
```
